# Genetic Structure and Relationship Analysis of an Association Population in Jute (*Corchorus* spp.) Evaluated by SSR Markers

**DOI:** 10.1371/journal.pone.0128195

**Published:** 2015-06-02

**Authors:** Liwu Zhang, Minhang Yuan, Aifen Tao, Jiantang Xu, Lihui Lin, Pingping Fang, Jianmin Qi

**Affiliations:** Key Laboratory of Ministry of Education for Genetics, Breeding and Multiple Utilization of Crops, College of Crop Science, Fujian Agriculture and Forestry University, Fuzhou 350002, China; National Institute of Plant Genome Research (NIPGR), INDIA

## Abstract

Population structure and relationship analysis is of great importance in the germplasm utilization and association mapping. Jute, comprised of white jute (*C*. *capsularis* L) and dark jute (*C*. *olitorius* L), is second to cotton in its commercial significance in the world. Here, we assessed the genetic structure and relationship in a panel of 159 jute accessions from 11 countries and regions using 63 SSRs. The structure analysis divided the 159 jute accessions from white and dark jute into Co and Cc group, further into Co1, Co2, Cc1 and Cc2 subgroups. Out of Cc1 subgroup, 81 accessions were from China and the remaining 10 accessions were from India (2), Japan (5), Thailand, Vietnam (2) and Pakistan (1). Out of Cc2 subgroup, 35 accessions were from China, and the remaining 3 accessions were from India, Pakistan and Thailand respectively. It can be inferred that the genetic background of these jute accessions was not always correlative with their geographical regions. Similar results were found in Co1 and Co2 subgroups. Analysis of molecular variance revealed 81% molecular variation between groups but it was low (19%) within subgroups, which further confirmed the genetic differentiation between the two groups. The genetic relationship analysis showed that the most diverse genotypes were Maliyeshengchangguo and Changguozhongyueyin in dark jute, BZ-2-2, Aidianyehuangma, Yangjuchiyuanguo, Zijinhuangma and Jute 179 in white jute, which could be used as the potential parents in breeding programs for jute improvement. These results would be very useful for association studies and breeding in jute.

## Introduction

Jute is a member of the *Corchorus* genus in the Tiliaceae family and is second to cotton in its commercial importance in the world. It is mainly distributed in China, India, Bangladesh and east-central Africa, where it has been grown for several thousand years for natural fiber production [1–2]. Since jute fiber is characterized as an environment-friendly, biodegradable and renewable cellulose fibre, it is called golden fiber.

Although there are nearly 60 species under the *Corchorus*, the commercially cultivated species are white jute (*C*. *capsularis*) and dark jute (*C*. *olitorius*), each of which is a diploid species (2n = 14) [1, 3]. The origin center of white jute is said to be Indo-Burma while the one of dark jute is Africa using morphological traits [1]. Recently, Kundu et al. (2013) suggested that the two cultivated species of jute originated in Africa using nuclear and chloroplast simple sequence repeats (SSRs) or microsatellites [4]. Although it is not certain how the two cultivated species of jute originated, they are different in terms of growth habitat, disease resistance, and characteristics associated to flowering and silique shape [1, 5]. For example, the silique shape of white jute is round, whereas the one of dark jute is long. In addition, the two cultivated species are cross-incompatible possibly due to the presence of a sexual incompatibility barrier [1]. Therefore, to improve jute fiber yield and quality using transgenic or cross-breeding methods, the divergent genotypes in the two cultivated species should be identified.

Currently, some studies on genetic diversity in jute have been carried out with morphological traits [6]. But this method is subject to environmental variation and time-consuming. This limitation could be overcome by using molecular markers, which are not influenced by the environment. Among various molecular markers, SSRs have become established as a power tool for genetic diversity studies because SSRs have several desirable characteristics, such as high level of reproducibility, codominant nature and abundance [4–5, 7–9]. Furthermore, SSR markers have high level of cross-species transferability for the two different jute species [2, 10]. Therefore, to date SSR markers have been utilized on genetic diversity in jute [4–5, 8–9]. Huq et al. [8] analyzed the genetic diversity in 16 jute genotypes using 27 SSRs and found that the alleles per locus of jute were 6.33. It was close to the finding of Ghosh et al. [5] using 6 polymorphic SSRs with 63 jute genotypes, but higher than that report by Banerjee et al. [9] involving 172 SSRs with 292 jute accessions. The difference of the alleles per locus among these previous studies may be caused by the differences of tested germplasms. Thereby, it can be seen that different germplasms might have different genetic backgrounds.

Although there were some genetic diversity studies carried out with SSR markers to assess the genetic variation in jute [4–5, 8–9, 11–12], few previous studies involved in the jute accessions from China, especially the local accessions. In recent years, the total yield of jute fiber in China, one of the main producers in the world, has slightly increased from 2003 to 2012 (http://www.jute.org/statistics_07.htm). And South China is said to be one of the origin centers of white jute [1]. The jute accessions from China had attracted attention because of their commercial importance and breeding value. Hence, additional research is imperative to conduct the genetic structure and relationship analysis of jute germplasm resources, especially involved in Chinese local accessions.

In the present study, we genotyped a panel of 159 jute accessions from 11 countries and regions, out of which originated from Chinese locations account for 81%, using 63 highly polymorphic SSR primers. The objectives of this research were to investigate the population structure and assess the genetic relationship among these jute germplasms.

## Materials and Methods

### Plant Materials

The 159 jute accessions, which were provided by Laboratory of Bast Crop Genetics and Breeding of Fujian Agriculture and Forestry University, are presented in S1 Table. Among them, 129 accessions were white jute (*C*. *capsularis*) and 30 accessions were dark jute (*C*. *olitorius*). These accessions, which included 129 accessions from China, 26 accessions from Bangladesh, India, Japan, Thailand, Vietnam and Pakistan, 3 accessions from Africa, and 1 accession from America, were selected on the basis of diverse geographical location for population structure analysis (Table 1). Two parental accessions, ‘Jute179’ and ‘Aidianyehuangm’ that had been used to develop a recombinant inbred lines (RILs) population for construction a SSR genetic map, were included in the panel of jute accessions.

**Table 1:** Distribution of 159 jute accessions from different countries among inferred groups and subgroups as defined by STRUCTURE.

Country	Co1	Co2	Total	Country	Cc1	Cc2	Total
China	2	11	13	China	81	35	116
Japan	0	1	1	Japan	5	0	5
India	0	2	2	India	2	1	3
Pakistan	0	4	4	Pakistan	1	0	1
Vietnam	0	2	2	Vietnam	2	0	2
Bangladesh	0	1	1	Bangladesh	0	1	1
Nepal	0	2	2	Thailand	0	1	1
Mali	1	1	2				
Kenya	0	1	1				
Cube	0	1	1				
Unknown*	0	1	1				
Total	3	27	30	Total	91	38	129

### DNA Extraction

All the 159 jute accessions were planted in 2013 in the experimental farm of Fujian Agriculture and Forestry University, Fuzhou, China. Genomic DNA from these jute accessions was extracted from 30-day-old seedlings using a modified cetyltrimethyl ammonium bromide method [10, 13] and preserved at -20°C. Before polymerase chain reaction (PCR), the DNA was diluted to the concentration of 50 ng/*μ*L with double distilled H_2_O.

### SSR Genotyping

SSRs prefixed with CcSSR and CoSSR were developed by our laboratory [10] and were used for genotyping. Sixty three SSR primers, which evenly distributed in different linkage groups, were selected for SSR genotyping (S2 Table). PCR reactions, electrophoresis and staining of PCR products were performed as described by Zhang et al. [10].

### Statistical Analysis

#### Population Structure Analysis

The amplified bands of SSRs were marked as present or absent. The population structure of 159 jute accessions was inferred using a program STRUCTURE v2.2 [14]. Because many accessions were selected from local regions, the classification of these germplasms was largely based on the results of STRUCTURE. The unrooted neighbor-joining (NJ) tree, on the basis of the Nei’s distance by MEGA 4.0 soft [15], was implemented to evaluate genetic relationship among these jute accessions.

#### Molecular Diversity Analysis

Polymorphism information content (PIC) and the number of alleles were estimated by PowerMarker 3.51 [16]. Analysis of molecular variance (AMOVA) was employed using Nei’s genetic distance matrix in Arlequin V3.1 [17].

#### Genetic Similarity Coefficient Analysis

Genetic similarity coefficient (GSC) comparing all pairs of the 159 jute accessions was calculated using unweighted pair group method of arithmetic means (UPGMA) by NTsys [18].

## Results

### Population Structure Analysis in the Panel of 159 Jute Accessions

**Fig 1:**
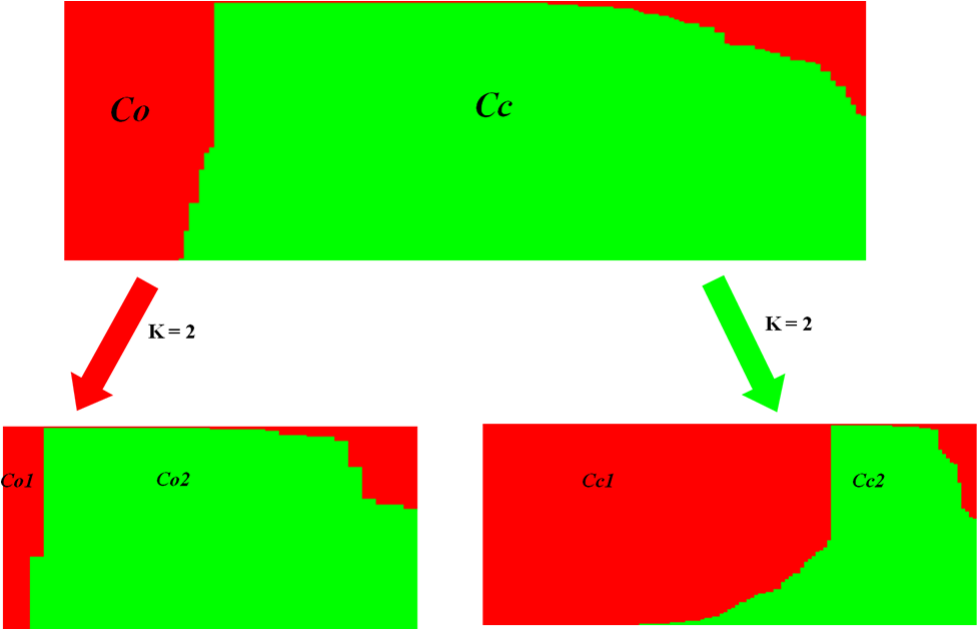
Population structure of 159 jute accessions based on 63 SSRs. When k (the number of subpopulations) is at 2, the 159 jute accessions from *C*. *olitorius* and *C*. *capsularis* were divided into two distinct groups, Co and Cc respectively. And when k = 2, the Co group were further divided into two subgroups, Co1 and Co2 respectively, and the Cc group were divided into two subgroups, Cc1 and Cc2 respectively.

Fig 1 and Table 1 show the population structure among the 159 jute accessions. Because a sharp peak of *D*_*k*_ appeared at k = 2 (S1 Fig), the 159 jute accessions were divided from white and dark jute into two distinct groups (Fig 1, Table 1), designated as Cc and Co respectively. Among the two distinct groups, the Cc group contained 129 white jute accessions, and the Co group contained 30 dark jute accessions. The classification of the 159 jute accessions from white and dark jute into Co and Cc group suggested that there was significant genetic variation between the two cultivated species of jute. Furthermore, the Cc and Co groups were subdivided into Cc1, Cc2, Co3 and Co4 subgroups respectively (S1 Fig). The Cc1 subgroup contained 91 accessions. Out of Cc1 subgroup, 81 accessions were from China, and the remaining 10 accessions were from India (2), Japan (5), Thailand, Vietnam (2) and Pakistan (1). The Cc2 subgroup contained 38 accessions, 35 of which were from China, and the remaining 3 accessions were from India, Pakistan and Thailand respectively. The Co1 subgroup contained 3 accessions, 2 of which were accessions from China, and the remaining 1 accession was from Mali. The Co2 subgroup contained 27 accessions, 11 of which were from China, and the remaining 15 accessions were from India (2), Japan (1), Vietnam (2), Pakistan (4), Bangladesh (1), Africa (4) and North America (1). From the location of different genotypes within subgroups, it can be inferred that population structure among these jute accessions might not be affected by genotypes of a particular location.

**Fig 2:**
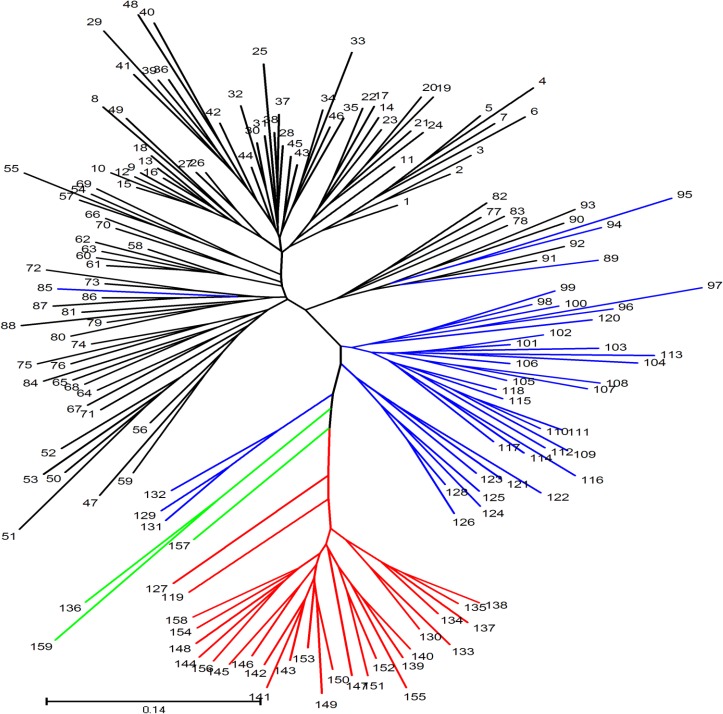
Unrooted Neigbour-Joining tree of 159 jute accessions. Green, red, black and blue lines correspond to assignments to different subgroups Co1, Co2, Cc1 and Cc2 respectively on the basis of the results of STRUCTURE analysis.

Similar results were also found using tree-based analyses (Fig 2). A total of 159 jute accessions assigned to different subgroups Co1, Co2, Cc1 and Cc2, which correspond to assignments green, red, black and blue lines, respectively.

### Analysis of Molecular Diversity among Inferred Groups and Subgroups

A summary of the genetic diversity parameters for each group and subgroup are listed in Table 2. The total of polymorphic bands was 211, with an average of 3.4 per primer. Although the sample size of Cc group was four times than that of Co group, there is no obvious difference between the alleles of Cc and Co, which were 198 and 172 respectively. The average locus diversity ranged from 0.037 to 0.770, and the average locus diversity is 0.46. The average polymorphism information content (PIC) value, which is a measure of heterozygosity in the test germplasms, was 0.33. The PIC value was relatively higher (0.35) in group Co than in group Cc (0.30) indicating the allelic diversity in dark jute was greater than that in white jute.

**Table 2:** Diversity in the jute groups and subgroups as defined by STRUCTURE.

Items	Total	Co^a^			Cc		
		Total	Co1^b^	Co2	Total	Cc1	Cc2
Sample size	159	30	3	27	129	91	38
Alleles	211	172	100	163	198	176	185
Gene diversity	0.46	0.35	0.43	0.32	0.42	0.40	0.40
PIC	0.40	0.30	0.35	0.28	0.35	0.34	0.34

^a^ Groups Co and Cc were classified on the basis of population structure analysis of the 159 jute accessions.

^b^ The Co group were further divided into Co1 and Co2 subgroups, and the Cc group into Cc1 and Cc2 subgroups.

Analysis of molecular variance (AMOVA) was conducted for partitioning the molecular variation at the group and subgroup levels (Table 3). The analysis of molecular variance (AMOVA) results showed that most of the molecular variance (79%) was distributed between groups (Co and Cc), not within subgroups (21%). Besides a strong sexual incompatibility barrier between the two cultivated species, it was possibly due to high selection pressure during crop improvement.

**Table 3:** Analysis of molecular variance among inferred groups and subgroups as defined by STRUCTURE.

Source of variation	DF^c^	SS ^d^	MS	Estimated variance	%
Among groups^a^	1	7918.68	7918.68	708.00	79
Among subgroups^b^	157	29509.71	187.96	187.96	21
Total	158	37428.39		895.05	

^a^ Groups were classfied by STRUCTURE analysis when K = 2, including Co and Cc.

^b^ Subgroups were separately defined within Co and Cc respectively.

^c^ Stands for the degree of freedom.

^d^ SS = Sum of squares; MS = Mean square.

### Genetic Similarity Coefficient Analysis among 159 Jute Accessions

To uncover the genetic difference of diverse genotypes, the GSC analysis among all pairs of the 159 jute accessions was conducted on the basis of structure analysis (Table 4). On the whole, the genetic variation of Co group is more than that of Cc group according to the comparison of coefficient variation of GSC. It might be related with the difference—the percentage of natural cross pollination of dark jute was a relatively higher than that of white jute.

**Table 4:** Genetic similarity coefficient in the jute groups and subgroups as defined by STRUCTURE.

Items	Co ^a^			Cc		
	Total	Co1^b^	Co2	Total	Cc1	Cc2
Sample size	30	3	27	129	91	38
Mean	0.750	0.420	0.771	0.711	0.711	0.719
Range	0.523–0.901	0.250–0.620	0.613–0.896	0.515–0.939	0.511–0.932	0.524–0.908
CV ^c^	11.18	44.48	6.87	8.98	8.87	8.95

^a^ Groups Co and Cc were classified on the basis of population structure analysis of the 159 jute accessions.

^b^ The Co group were further divided into Co1 and Co2 subgroups, and the Cc group into Cc1 and Cc2 subgroups.

^c^ Coefficient of variation

Out of Co group, the GSC ranged from 0.523 to 0.901, with the average of 0.750 (Table 4; S3 Table). The pairs of GSC which was above 0.88 were Bama 71 and Dianbianqingma, Bachang No. 4 and Dianbianqingm, Bachang No. 4 and Bama 71, Putianqingma and Guangfengchangguo, and so on. Of them, Bachang No. 4 and Kuanyechangguo had the highest GSC (0.97), indicating a close interrelationship between them. According to the pedigree analysis, Kuanyechangguo is a pure cultivar selected from a cross between Bachang No. 4 and Guangfengchangguo. Out of 30 dark jute accessions, Maliyeshengchangguo is worth mentioning because the lowest average GSC was observed (0.594). The pair of the lowest GSC (0.48) is Maliyeshengchangguo and Cuilv. It indicated that these accessions, like Maliyeshengchangguo and Changguozhongyueyin (0.599), could be used as the potential parents in future breeding programs for dark jute improvement.

Out of Cc group, the GSC ranged from 0.511 to 0.932, with the average of 0.711 (Table 4; S4 Table). Among the all pairs of 129 white jute accessions, the GSC of some pairs was high. For example, the GSC of the pairs of Meifeng No. 1 and Meifeng No. 2, Yueyuan No. 2 and Yueyuan No. 4, Shaowuhuangma and C46 were above 0.90. According to the pedigree analysis, most of them are sister-lines. The GSCs of some pairs were low. The pairs, whose GSC were below 0.54, were BZ-2-2 and Yueyuan No. 6, BZ-2-2 and Wuma 1, Yangjuchiyuanguo and Wuma 1, Aidianyehuangma and Minhouhongpi, Jute 179 and Minhouhongpi, Zijinhuangma and Jinshanhuangma, and so on. Among them, BZ-2-2 is worthy to mention because the lowest average GSC was observed. All these results provide potential useful parents, like BZ-2-2, Aidianyehuangma, Yangjuchiyuanguo, Zijinhuangma and Jute 179, for white jute improvement.

## Discussion

Since SSRs can be easily detected by PCR, SSRs have been widely used in the analysis of population structure and genetic diversity in jute [5, 8, 9]. In the present study, we assessed the population structure of 159 genotypes using 63 SSRs, which evenly distributed linkage groups in jute. From their geographical regions for cultivation, 81 percent of the 159 jute accessions originated from Chinese location varieties, which is quite different from the previous studies [11–12, 19]. On the basis of the structure analysis, the 159 accessions from white and dark jute were divided into two distinct groups (Co and Cc), which indicated that significant genetic variation existed in the two cultivated species of jute. This pattern of classification is in agreement with the results in previous studies [11–12, 19]. The difference between the two cultivated species might be due to the different origin centers of each species. As reported by Xiong [1], dark jute possibly originated from Africa while white jute originated from the India and South China region. The fact of strong sexual incompatibility barrier between white and dark jute further confirmed it [1, 20]. However, it was also observed that a few accessions in each subgroup that are not in agreement with their geographical origins. That is, the genetic background of the 159 jute accessions was not always correlative with their geographical regions. This phenomenon might be due to germplasm exchange across boundaries. The above results suggest that it is very useful to conduct population structure analysis among a panel of 159 jute accessions, out of which originated from Chinese locations account for 81%.

The genetic diversity parameters for each group suggested that there is relatively much genetic differentiation between the two groups (Cc and Co). This was confirmed by AMOVA, where low level of estimated variation (21%) observed within subgroups compared to four fold (79%) higher variation within groups (Table 3). It also can be confirmed by the results of tree-based analysis and population structure separately (Fig 1 and Fig 2). These data suggest that there is a higher level of genetic diversity in dark jute than in white jute. The results of genetic diversity in jute by Banerjee et al. [9] and Ghosh et al. [5] are in accord with this finding. A higher level of genetic diversity may indicate a higher level of natural outcrossing in plants. As reported by Xiong [1], the percentage of natural cross pollination of dark jute (8–12%) is a relatively higher than of white jute (3–4%). However, the genetic diversity parameters for each subgroup suggested that there was relatively poor genetic difference among the genotypes of subgroup. It can be reflected by the estimates of variation coefficient of GSC. In white jute, coefficient variation of GSC in Cc1 was similar to that in Cc2. This might be a result of germplasm exchange between geographical regions for cultivation. Meanwhile, we observed that coefficient variation of GSC was much higher in Co1 than in Co2 in dark jute (Table 4) indicating that the genotypes in Co1 have a considerable amount of genetic diversity. This was possibly due to the lowest average GSC (0.594) of Maliyeshengchangguo which is originated from Mali in Africa.

As is known, parental accessions of obvious genetic background difference usually generates an elite variety in the cross-breeding program. Thus, understanding of genetic relationship is critically important for crop improvement [1, 21]. The results of GSC (Table 4) showed that the coefficient of variation of GSC from Co1 (44.48%) was extraordinary diversified among the four different subgroups, that from Co2 (6.873%) was the least, and from Cc1 and Cc2 were placed in the middle (8.87% and 8.95% respectively). To broaden the genetic variation, it is essential to conduct inter-subgroup cross-breeding programs in jute. In this study, the most divergent genotypes—namely, Maliyeshengchangguo and Changguozhongyueyin in dark jute and BZ-2-2, Aidianyehuangma, Yangjuchiyuanguo, Zijinhuangma and Jute 179 in white jute—were identified in GSC analysis, which could be used in cross-breeding programs for jute improvement. Moreover, advanced technologies, such as somatic hybridization and genetic transformation, could be used to overcome the sexual incompatibility barrier between dark and white jute.

## Supporting Information

S1 Fig Line chart of K value changing with ΔK in the total panel and inferred groups in 159 jute accessions. a) the total panel; b) the Co group; c) the Cc group.
(TIF)

S1 Table List of 159 jute accessions and population assignment based on STRUCTURE analysis. (XLS)

S2 Table Information of 63 SSR primers in this study. (XLS)

S3 Table Genetic similarity coefficient among inferred subgroups (Co) as defined by STRUCTURE. (XLS)

S4 Table Genetic similarity coefficient among inferred subgroups (Cc) as defined by STRUCTURE. (XLS)
