# Supplementary figures and images for: Genetic Structure and Relationship Analysis of an Association Population in Jute (Corchorus spp.) Evaluated by SSR Markers
Source: PLoS One. 2015 Jun 2;10(6):e0128195. doi: 10.1371/journal.pone.0128195 (PMC4452778; doi:10.1371/journal.pone.0128195)

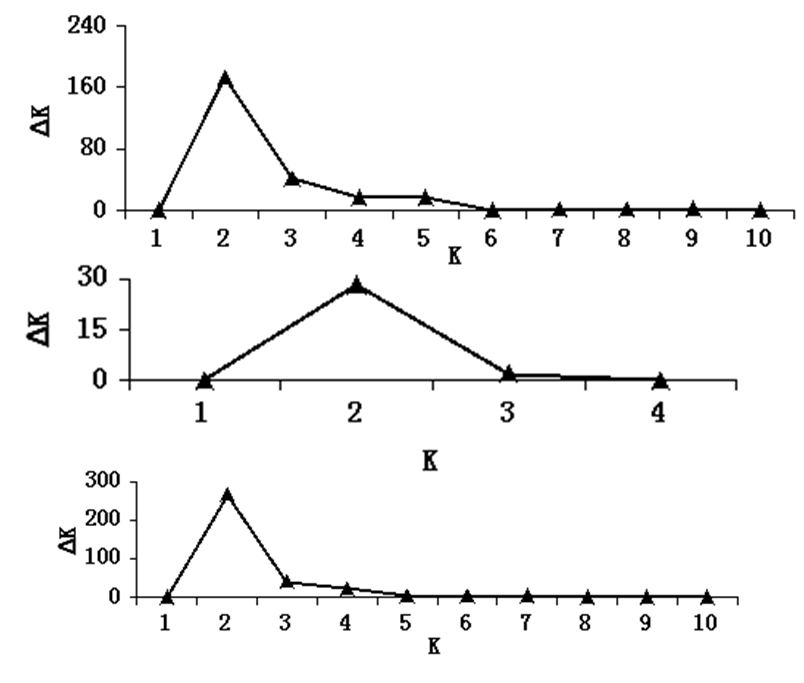

Supplement: S1 Fig — a) the total panel; b) the Co group; c) the Cc group. (TIF) [file pone.0128195.s001.tif]
